# P344: Situational analysis of the organization and implementation of quality standards of care in Liberia

**DOI:** 10.1186/2047-2994-2-S1-P344

**Published:** 2013-06-20

**Authors:** N Dunbar

**Affiliations:** 1 Department Planning/Research & Man Power Development, Ministry of Health and Social Welfare, Monrovia, Liberia

## Introduction

A study on situational analysis of quality health care norms and standards was conducted to support MOH/SW, WAHO and RIPAQS initiative in the development of an institutional and regulatory framework to support and promote the quality management process and evaluation of professional practices in national or even regional health institutions in Africa.

## Objectives

The study focused on the evaluation of quality management in health systems, collection of information on the existence of policies in quality health care standards and norms, determining health workers knowledge, attitudes and practice on best practices recommendation to contribute to strengthening strategies aimed at sustaining quality health care management and setting up norms and standards for professional practices in health care institutions in West Africa.

## Methods

The study was a multidisciplinary study and prospective evaluation of national development strategies of quality health in national health systems, with a an idea of assessing patientsafety to evaluate and make an inventory of the quality approach and application of those standards and quality health care norms;and safety culture in nine (9) health institutions (Secondary and Primary facilities) in Liberia.

## Results

A total of 334 health professionals in nine (9) health facilities (Redemption Hospital, James Davids Hospital, St. Joseph Catholic Hospital, ELWA Hospital, Soniwein Health Center, Barnesville Health Center, Clara Town Health Center, Duport Road Health Center and People United Community Clinic) with various specialties were interviewed. All nine (9) facilities assessed are providing quality health care to some level as, there are some good performances by health facilities in terms of provision of quality health services in institutional, technical and operational dimensions. Most health facilities are practicing at least some form of quality health care best practices; however, there are gaps that need to be bridged for the provision of quality health care standards and norms.

## Conclusion

The provision of quality health care standards and norms in the Liberia health system requires strengthening at all levels of service delivery in the institutional, technical and operational dimensions of quality health care.

## Disclosure of interest

None declared

